# Th17 Cells in Periodontitis and Its Regulation by A20

**DOI:** 10.3389/fimmu.2021.742925

**Published:** 2021-09-07

**Authors:** Ning Huang, Hao Dong, Yuqi Luo, Bin Shao

**Affiliations:** State Key Laboratory of Oral Diseases & National Clinical Research Center for Oral Diseases, West China Hospital of Stomatology, Sichuan University, Chengdu, China

**Keywords:** T helper cells, Th17, inflammation, periodontitis, A20 (TNFAIP3)

## Abstract

Periodontitis is a prevalent chronic disease that results in loss of periodontal ligament and bone resorption. Triggered by pathogens and prolonged inflammation, periodontitis is modulated by the immune system, especially pro-inflammatory cells, such as T helper (Th) 17 cells. Originated from CD4^+^ Th cells, Th17 cells play a central role for they drive and regulate periodontal inflammation. Cytokines secreted by Th17 cells are also major players in the pathogenesis of periodontitis. Given the importance of Th17 cells, modulators of Th17 cells are of great clinical potential and worth of discussion. This review aims to provide an overview of the current understanding of the effect of Th17 cells on periodontitis, as well as a brief discussion of current and potential therapies targeting Th17 cells. Lastly, we highlight this article by summarizing the causal relationship between A20 (encoded by *TNFAIP3*), an anti-inflammatory molecule, and Th17 cell differentiation.

## Introduction

Periodontitis, influencing nearly 10%-15% people globally, is a common chronic disease featured by periodontal inflammation and alveolar bone destruction (1, 2). The pathogenesis of periodontitis mainly involves disease-associated oral microbiota, host inflammation, as well as environmental and genetic risk factors (3, 4). Ultimately, chronic and overwhelming inflammation causes periodontitis, which could only be terminated by tooth loss or therapeutic interventions. Recent studies have suggested a correlation between periodontitis and other systemic diseases including obesity, diabetes, hypertension, cardiovascular diseases and Alzheimer’s disease (5–9).

T cells are positioned critically in the pathogenesis of periodontitis, especially Th17 cells. Th17 cells are a lineage of CD4^+^T cells that are known for producing proinflammatory cytokine interleukin (IL)-17 (10). Induced by retinoid-related orphan nuclear receptor γt (RORγt, in man-made homologue RORC), Th17 cells recruit neutrophils, regulate chemokine receptors, initiate inflammation and bone resorption through pro-inflammatory cytokines such as IL-6, IL-17, IL-23 (11–13). Th17 cells also exaggerate inflammation further by recruiting Th17 cells (14, 15). Although Th17 cells act against microbial signals, especially in mucosal immunity, Th17 cells are inflammation enhancers that promote periodontal inflammation and bone resorption in the oral cavity (16). Accordingly, antibodies targeting IL-17 have protected mice from severe periodontitis, including diabetic ones (17, 18). Also, modulators of the Th17/T regulatory (Treg) balance, such as boldine, IL-35, IL-10 secreting B cells, calcitriol, showed efficacy in alleviating periodontitis (19–22). Beyond the oral cavity, IL-17 blockade also showed promising outcomes in treating immune diseases like psoriasis (23).

To restrict excessive inflammation in the oral cavity, the immune system has developed a set of restricting measures to attain timely termination of inflammation and maintain the holistic equilibrium. For example, the ubiquitination system modulates intracellular homeostasis through covalent enzymatical post-transcriptional modifications. Ubiquitin-editing enzymes activate, conjugate, ligate or remove polyubiquitin chains from their substrates enzymatically, thus deciding protein fate and regulating immune responses (24). A20 (TNFAIP3) is a ubiquitin-editing enzyme that has established its role as a potent anti-inflammatory molecule. A20 deubiquinates key factors of nuclear factor-κB (NF-κB), thereby blocking NF-κB pathway and arresting immune responses (25, 26). On the N-terminal of A20, ovarian tumor (OTU) domain enables A20 to deubiquitinate (27). While on its C-terminal, seven zinc finger (ZnF) domains confer A20 with ubiquitin-binding ability (28). In recent years, A20 displayed pleiotropic effects in cell death, tumorigenesis and autoimmune diseases (29–31). Hereinbelow, we discuss the regulatory role of A20 in Th17 cell differentiation and IL-17 function.

In this review, we summarized the pivotal role of Th17 cells in periodontitis as well as their modulation by cytokines and transcription factors. We also concluded the heterogeneity and plasticity of Th17 cells. Current therapies targeting Th17 cells are summarized as well. Moreover, as a team that focus on A20-related studies, we highlight this article by concluding the negative effect of A20 on Th17 cell expansion and IL-17 signaling, which could be a potential tool in treating periodontitis.

## Dysbiosis and Dysregulated Inflammation Drive Periodontitis

The understanding of the aetiology of periodontitis has gone through different stages. Originally, it was thought to be a rather simple bacterial infection attributed to a small group of bacteria. However, later studies revealed the existence of influential factors other than microbiota, for example host reaction and environmental factors (3, 32–34).Put simply, the pathogenesis of periodontitis starts from gingivitis. The synergy between dysbiosis (changes or imbalance in the composition and abundance of oral microbial communities) and aberrant immune responses is the exact reason why gingivitis develops into periodontitis (35).

**Figure 1:**
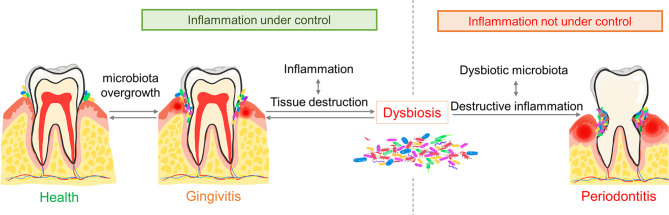
The pathogenesis of periodontitis. The overgrowth of commensal microbiota triggers gingivitis. Gingival inflammation then provides nutrients and a local anaerobic environment for periodontal pathogens. The overgrowth and diversity of periodontal pathogens exaggerate inflammation which decides the progression of gingivitis. Dysregulated inflammation together with microbial dysbiosis lead to periodontitis. Conversely, balanced inflammation leads to quiescent gingivitis.

Physiologically, the oral cavity is always in a delicate balance between local immune activation and suppression (36, 37). However, under certain circumstances, the overgrowth of oral commensal microbiota leads to gingivitis, a destructive but reversible inflammatory disease. Then, depending on host susceptibility, some patients may suffer from the conversion to periodontitis, while others maintain long-term stability (38). In susceptible hosts, sustained gingival inflammation forms inflamed pockets in which gingival crevicular fluid provides essential nutrients (including abundant collagen decomposition products, serum exudates, etc.) for bacteria (34, 39). In addition, such inflammatory conditions create an anaerobic environment where anaerobic bacteria proliferate, finally leading to the overgrowth of commensal microbiota and dysbiosis (40, 41). Dysbiosis exacerbates inflammation and conversely, chronic dysregulated immune responses in turn facilitate dysbiosis by providing tissue decomposition as nutrients for bacteria, thus forming a positive feedback loop (35, 40) (**Figure 1**).

Of note, periodontitis could be independent of microorganisms. It was reported that germ free (GF) mice did not present alveolar bone loss even with *P. gingivalis* infection (42). Also, a combined application of therapeutic strategies targeting inflammatory response achieved better results than simple plaque removal, revealing the key role of host response in periodontal tissue destruction (43–46). Besides dysbiosis and dysregulation of immune responses, genetic and environmental factors are also crucial in the pathogenesis of periodontitis. Genetic polymorphisms may increase the risk of inflammatory disease varied by region and race/ethnicity (47). A recent study reported that in the Asian population, tumor necrosis factor (TNF)-α G-308A (rs1800629) polymorphism is linked with increased susceptibility to chronic periodontitis (48). Also, smokers are at least 50% more likely to develop periodontitis than non-smokers, with faster progression, severer deterioration, and poorer treating efficacy (49, 50). Weight gain may also be one of the risk factors for periodontitis, for clinical evidence suggests that obese people have a higher risk of periodontitis (51). Long-term psychological stress or anxiety leads to both worse periodontal conditions and a negative impact on the effectiveness of periodontal treatment (52, 53). In summary, periodontitis is a multifactorial chronic inflammatory disease mainly caused by dysbiosis, dysregulated immune system along with genetic and environmental factors.

## Th17 Cells at a Glance: Beyond the Th1/Th2 Paradigm

Currently, Th cells are commonly categorized into five major subsets: Th1, Th2, Th17, T-follicular helper (Tfh) and Treg cells (54). All these CD4^+^ T cells play important roles in host immune defence against harmful microorganisms as well as in inflammation diseases (54, 55). Here we will give a brief introduction on the discovery of Th17 cells.

In 1986, Mosmann and Coffman pioneered the classification of CD4^+^ T cells into two subsets: Th1 cells and Th2 cells (56). Immature CD4^+^ T cells differentiate into specific lineages of Th cells under the regulation of local cytokine milieu and transcription factors. IL-12 and interferon (IFN)-γ activate transcription factors signal transducer and activator of transcription (STAT)1, STAT4 and T-bet in CD4^+^ T cells, which favor the differentiation into Th1 cells (57, 58). Similarly, IL-2 and IL-4 promote the differentiation of Th2 cells by increasing the expression of STAT6 and GATA-3 (59, 60). The classic Th1/Th2 paradigm preliminarily reveals the diversity of CD4^+^ T cells in function. Th1 cells defend against intracellular organisms while Th2 cells targets extracellular pathogens (61, 62).Abnormal activation of Th1 cells and Th2 cells is also a key factor in the pathogenesis of many autoimmune diseases and inflammatory reactions.

The study of Th1/Th2 paradigm has helped to understand the pathogenesis of many diseases, including multiple sclerosis, psoriasis and so on (63, 64). However, a study on experimental autoimmune encephalomyelitis (EAE) raised certain doubts. EAE is a disease previously attributed to Th1 cells but blockade of Th1 cells failed to protect mice from disease progression (65). Consequent studies discovered a novel lineage of CD4^+^ T cells characterized by IL-17 production, beyond the Th1/Th2 paradigm (66–68). H. Park et al. and L. E. Harrington et al. pioneered the discovery of a new pedigree of Th17 cells, which filled some gaps in host immune response and the pathogenesis of autoimmune diseases (10, 69). Th17 cell subsets were originally named after their main cytokine IL-17A. IL-17A also comes from γδT cells, natural killer T (NKT) cells and congenital lymphoid cells after sensing pathogen invasion or injury signals (70, 71). These cells are collectively called type 17 cells and are characterized by the expression of RORγt and IL-23R (11, 72). Th17 cells act as important defenders against pathogen invasion, especially fungal infections. This explains why patients with congenital defects of Th17 cells have higher susceptibility to fungal infections such as *Candida albicans (* 73). Also, considerable studies have shown that Th17 cells play a pivotal role in immune-mediated inflammatory diseases, including periodontitis, psoriasis, rheumatoid arthritis and so on (74–76).

## Th17 Cell Involvement in Periodontitis Pathogenesis

T cells are the major immune cell population in oral mucosal compartments both in health and disease. In periodontitis, the level of Th17 cells rockets, which indicates a close relationship between Th17 cells and periodontitis (77, 78). However, the complex role of Th17 cells and IL-17 in periodontitis is still controversial. Yu et al. found that IL-17RA^KO^ mice exhibited severer alveolar bone loss due to compromised chemokine expression and neutrophil migration (79). While Dutzan et al. reported the opposite conclusion in *Cd4^cre^Stat3^fl/fl^* and *Lck^cre^Rorc^fl/fl^* mice that defection in Th17 cell differentiation exhibited significantly reduced alveolar bone resorption compared with wildtype (17). IL-17 also displayed gender-dependent effect as female mice are more susceptible to alveolar bone loss due to impaired *P. gingivalis*-specific antibody response and chemokine production (80). Also, patients with autosomal dominant high IgE syndrome (AD-HIES, present with congenital poor Th17 cell differentiation) showed reduced susceptibility to periodontitis and less alveolar bone resorption (81, 82).

**Figure 2:**
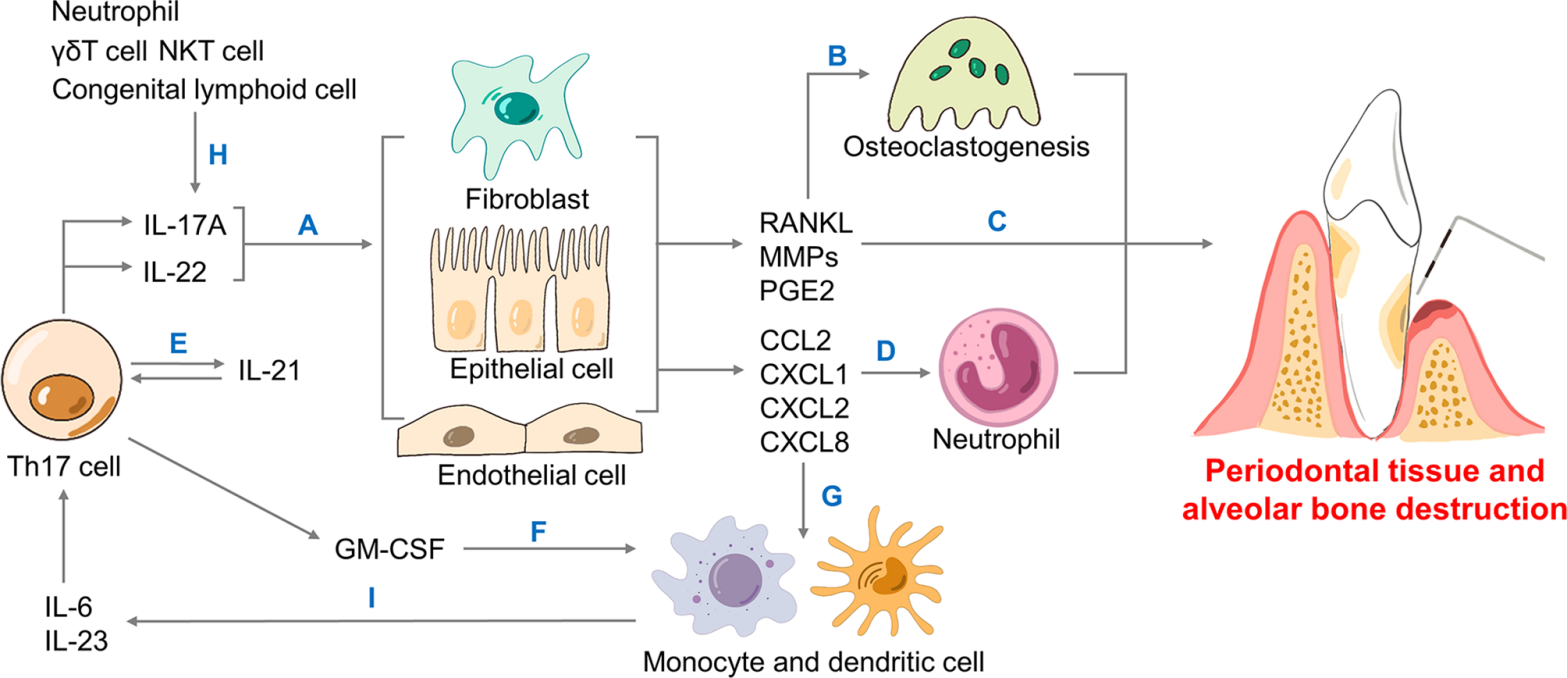
The role of Th17 cells in periodontitis. Th17 cells secrete cytokines including IL-17A, IL-22, IL-21 and GM-CSF. **(A)** IL-17A and IL-22 bind to non-hematopoietic cells like fibroblasts, epithelial and endothelial cells to generate RANKL, MMPs, PGE2, as well as chemokines. **(B)** RANKL leads to osteoclastogenesis, the driving force of bone resorption. **(C)** MMPs, PGE2 together with neutrophils contribute to periodontal inflammation and alveolar bone loss. **(D)** IL-17A mediates neutrophil recruitment in periodontitis *via* generating chemokines including CXCL1, CXCL2 and CXCL8. **(E)** Produced by Th17, IL-21 forms a positive loop that directly promotes Th17 recruitment. **(F)** Th17 cells produce GM-CSF that promotes monocyte and dendritic cells. **(H)** Neutrophils and innate type 17 cells like γδT cells, NKT cells and congenital lymphoid cells could induce IL-17A as well. **(G)** Chemokines contribute to monocyte and dendritic cells. **(I)** Monocytes and dendritic cells generate IL-6 and IL-23 that help Th17 cells differentiate. Th, T helper; IL-, interleukin-; GM-CSF, granulocyte-macrophage colony-stimulating factor; RANK, receptor activator for nuclear factor-κB; RANKL, RANK ligand; MMPs, metalloproteinases; PGE2, prostaglandin E2; CXCL, CXC motif ligand; NKT, natural killer T.

Hereinbelow, we discuss how Th17 cells promote periodontal inflammation and bone resorption through the secretion of IL-17A, IL-17F, IL-21, IL-22 and granulocyte-macrophage colony-stimulating factor (GM-CSF), as well as the interaction between Th17 cells with other immune cells (12, 16, 83–85) (**Figure 2**). Besides, *P. gingivalis* further amplifies such inflammation-mediated destruction.

### The Functions of Molecules Secreted by Th17

#### IL-17A

IL-17A is considered the main cytokine in the pathogenesis of periodontitis. Although it has a limited ability to induce inflammation directly, IL-17A could exert powerful inflammatory effects through synergistic effects with other inflammatory factors (86–88). IL-17A acts on non-hematopoietic cells such as fibroblasts, epithelial cells and endothelial cells to promote the expression of many inflammatory cytokines, including IL-1β, IL-6, IL-8, granulocyte colony-stimulating factor (G-CSF), GM-CSF and TNF-α (10, 83). Meanwhile, IL-17A upregulates the expression of C-C motif ligand (CCL) 2, C-X-C motif ligand (CXCL) 1, CXCL2, CXCL5 and CXCL8 (88).

**Figure 3:**
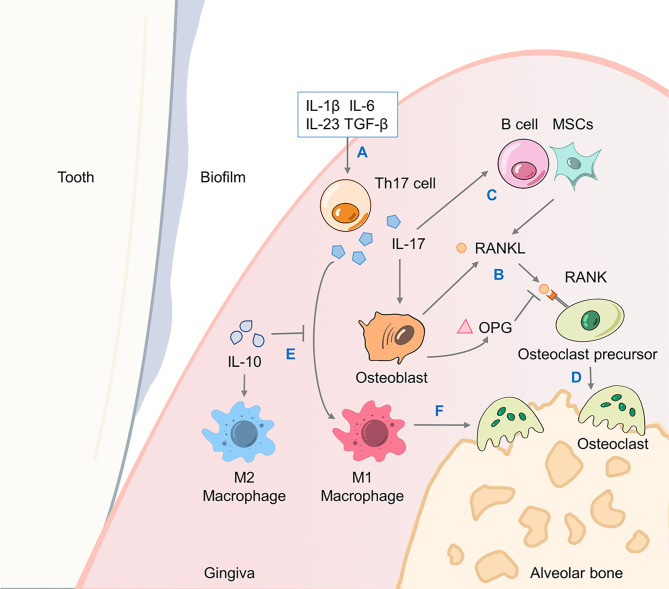
IL-17A drives alveolar bone resorption through the RANKL/OPG axis. The promotion of bone resorption by Th17 cells involves the joint action of a triad of proteins including RANKL, its functional receptor RANK and its decoy receptor OPG. **(A)** Cytokines including TGF-β, IL-1β, IL-6, IL-23, etc. promote the differentiation of Th17 cells and production of IL-17A. **(B)** Osteoblasts generate RANKL to bind to its receptor RANK on osteoclast precursor cells, as well as OPG to antagonize RANKL. **(C)** IL-17A-induced B cells and MSCs activation also promote RANKL production. **(D)** Osteoclast precursor cells differentiate and fuse into mature osteoclasts that causes alveolar bone resorption. **(E)** IL-17A mediates a proinflammatory M1 macrophage response which would be inhibited by IL-10, while IL-10 promotes M2 macrophage polarization. **(F)** M1 macrophages induce the production of proinflammatory cytokines such as IFN-γ and IL-6 to exacerbate alveolar bone loss. OPG, osteoprotegerin; TGF-β, transforming growth factor-β; MSC, mesenchymal stem cell; IFN, interferon.

IL-17A further recruits more neutrophils and monocytes through these chemokines and enhances their survival and activity by releasing more GM-CSF (16, 88). Furthermore, as inducers of human Th17 cell differentiation, IL-1β and IL-6 cooperate with IL-17A to form a positive feedback loop that enhances the inflammatory effect of IL-17A (89, 90). Finally, IL-17A could directly promote the destruction of periodontal connective tissue and alveolar bone by inducing the production of prostaglandin E2 (PGE2), matrix metalloproteinases (MMPs) and NF-κB receptor activator ligand (RANKL) (16). RANKL has a fundamental role in alveolar bone destruction, as the binding of RANKL to its functional receptor NF-κB receptor activator (RANK) on the precursor of osteoclasts could promote the maturation and activation of osteoclasts. The decoy receptor osteoprotegerin (OPG) competes against RANKL and binds to RANK, thus inhibiting osteoclast differentiation and bone resorption (**Figure 3**) (87).

The IL-17A signalling pathway initiates a cascade of inflammation. Specifically, IL-17A acts as a ligand that binds to the IL-17 receptor complex (IL-17RA/IL-17RC) and recruits the binding protein Act1 through the SEF/IL-17R (SEFIR) domain of the tail conserved domain on IL-17R (91). Act1 contains a tumour necrosis factor receptor-associated factor (TRAF) binding motif and possesses E3 ligase activity, meaning it could recruit and ubiquitinate TRAF6. After TRAF6 activation, transforming growth factor β (TGF-β) activated kinase (TAK) 1 and inhibitor of NF-κB (IκB) kinase (IKK) complexes were recruited and activated, which trigger NF-κB and mitogen-activated protein kinase (MAPK) pathways, eventually promoting the expression of inflammatory mediators and the activation of osteoclasts (92, 93). At the same time, IL-17RA possesses a unique C-terminal activation domain, called CCAAT/enhancer binding protein β (C/EBP β) activation domain (CBAD) that participates in the activation of transcription factor C/EBP β. C/EBP β not only mediates and enhances the synergistic effect of IL-17 and TNF signal, but also up-regulates the expression of inflammatory mediators such as IL-6 (94, 95).

#### IL-21

IL-21, a member of the IL-2 cytokine family, is another cytokine secreted by Th17 cell, but its exact role is incompletely understood. In some experiments, the level of IL-21 in the serum and saliva of patients with chronic periodontitis significantly increased, and the level of IL-21 is down-regulated after periodontal treatment, suggesting that IL-21 may promote periodontitis (96, 97). In the absence of IL-6, the synergistic effect of IL-21 and TGF-β activates STAT3 to promote the development of Th17 cells, and inhibits the expression of Forkhead Box P3 (FOXP3) (98, 99). However, some studies suggested that IL-21 plays a dispensable role in riving the inflammatory effect of Th17 cells because IL-21- and IL-21R-deficient mice were still highly susceptible to EAE (100, 101). Lastly, a specific study, in which IL-21 induces IL-10 expression in B10 cells and results in less alveolar bone loss, suggested that IL-21 inhibits inflammation (102). More experiments are needed to examine whether IL-21 is the driver/inhibitor/bystander of periodontitis.

#### IL-22

IL-22 is a member of the IL-10 cytokine family. IL-22 receptor is composed of the IL-22R1 subunit and the IL-10R2 subunit, shared with IL-10. Even though IL-10 downregulates pro-inflammatory cytokine expression, IL-22 often fails to do so. This is mainly because that the expression of human IL-22R1 is often limited to epithelial cells and endothelial cells, while immune cells usually lack the expression of IL-22R1 (103).

IL-22 is pro-inflammatory as it enhances the effects of co-acting pro-inflammatory factors such as TNF-α and IL-17 (104, 105). Specifically, IL-22 binds with IL-22R1 to form a complex that binds to IL-10R2, which usually activates the Janus kinase (JAK)-STAT signaling pathway, especially STAT3 as the main signal transduction pathway (106). In addition, IL-22 could also directly promote the expression of inflammatory mediators like MMP-1, resulting in connective tissue destruction and bone resorption (107, 108). Recent studies have revealed a positive correlation between the IL-22, RANKL expression and the severity of periodontitis (109, 110).

#### GM-CSF

GM-CSF acts on dendritic cells (DCs) and monocytes to promote the production of inflammatory factors such as IL-6, IL-23, facilitating differentiation of Th17 cells (111). Particularly, IL-23 in turn promotes GM-CSF production and forms a vicious cycle (112). Recent studies have shown that the role of GM-CSF in inflammation and bone loss may be underestimated. In a mouse model of experimental autoimmune uveitis (EAU), GM-CSF-driven eosinophil inflammation dominates the development of EAU without IL-17 and IFN-γ (113). GM-CSF expression is downregulated at the presence of IL-17A, whereas IL-17A deficiency would lead to the upregulation of GM-CSF and inflammatory reaction (114). Further experiments confirmed that IL-17A induced IL-24 through autocrine pathway, downregulating IL-17F and GM-CSF expression through suppressor of cytokine signalling (SOCS)1 and SOCS3 (114). The rise of GM-CSF may explain, to some extent, why the use of anti-IL-17A monoclonal antibodies alone aggravates symptoms seen in inflammatory bowel disease (IBD) (115).

### Synergistic Effects of Th17 With Other Immune Cells

#### Neutrophils

Neutrophils are now considered the vital cellular regulator of Th17 response in periodontitis. Excessive neutrophil accumulation in periodontium provokes inflammation, as the number of neutrophils is positively correlated with the severity of periodontitis (116–118). Neutrophils not only release reactive oxygen species (ROS) and MMPS to directly damage connective tissue, but also interact with adaptive immune cells, especially Th17 cells, to induce bone resorption (119, 120). Chemokine CCL2 and CCL20 produced by neutrophils recruit Th17 cells and facilitate their chemotaxis to the inflamed sites (121). IL-17, induced by neutrophils, also cooperates with IL-1β to increase the expression of CCL20 in human gingival fibroblasts and further recruit Th17 cells (122). As mentioned before, Th17-induced GM-CSF recruit neutrophils as well, eventually forming a feedback loop that leads to mutual recruitment.

Under physiological conditions, mutual recruitment ends up as the inflammation subsides. Phagocytosis of apoptotic neutrophils could inhibit the expression of IL-23 of phagocytes and then down-regulate the production of IL-17 and G-CSF, so that neutrophils decrease concomitantly with the regression of inflammation (123). Intriguingly, patients with leukocyte adhesion deficiency type I (LAD-I) have impaired leukocyte function but they still have a higher risk of periodontitis. The underlying mechanism could be the breakdown of the neutrophil regulation feedback circuit. Due to the lack of inhibition of IL-23 expression in LAD-I patients, excessive accumulation of IL-17 in periodontium eventually led to alveolar bone injury (124).

#### APCs

Antigen-presenting cells (APCs) present antigens and drive differentiation of Th cells, linking the innate immune response to the adaptive immune response (125, 126). Previous studies have demonstrated that periodontal pathogens stimulate APCs to upregulate the expression of markers that facilitate the proliferation of Th17 cells (122, 127). DCs principally mediate the adaptive immune response in periodontitis. Monocytes and macrophages are involved in instructing Th17 cell differentiation as well. Although their concentrations in healthy gingiva are relatively low, APCs increase significantly in patients with periodontitis (128).

Monocytes recognize *P. gingivalis* through toll-like receptors (TLR)2/4, and then upregulate the expression of IL-1β and IL-23 to induce Th17 cell differentiation (127). Also, Delta-like ligand 4 (Dll-4) expression in monocytes is upregulated by *P. gingivalis* lipopolysaccharide (LPS) to promote Th17 cell response (129). Periodontal pathogens also upregulate CD86 expression and induce monocyte differentiation into macrophages (127). As for macrophages, LPS- and IFN-γ-activated macrophages were induced by CCL21 (which shows up-regulation in periodontitis tissues). These macrophages elevate the expression of both CCR7 and cytokines such as IL-6 and IL-23, which drive the differentiation of naive T cells into Th17 cells and enhance osteoblast production (130, 131).

### Induction and Enhancement of Th17 Cell by *P. gingivalis*

*P. gingivalis* is a gram-negative anaerobe which has been implicated as a keystone pathogen that contributes to periodontitis (132). Previous studies suggested that *P. gingivalis* interacts with APCs to induce Th17 cell differentiation (126, 127). It has been demonstrated that the interaction between *P. gingivalis* and host DCs induces the production of a series of inflammatory factors including IL-17, IL-1β, IL-6, IL-23 etc., which enhances and stabilizes the differentiation of Th17 cells (126). In addition, independent of APC activation *in vitro*, *P. gingivalis*-LPS promotes Th17 cell differentiation directly through TLR2 signalling. It also enhances IL-17-mediated bone resorption by the up-regulation of transcription factors such as RORC as well (133, 134).

Differed in their ability to induce Th17 cell differentiation, *P. gingivalis* can be classified into virulent (*P. gingivalis* W83) and avirulent strains (*P. gingivalis* ATCC33277) (135). Compared with avirulent strains, LPS from virulent strains showed a higher induction of IL-1β and IL-6 as well as higher expression of RORC and IL-17, promoting Th17 cell differentiation more effectively (126, 134).

## Regulators of Th17 Cells Differentiation

### Regulatory Roles of Cytokines and Transcription Factors

Similar to that of Th1/2 cells, the differentiation of Th17 cells is induced by the synergistic action of STAT3 and RORγt which are regulated by local environment and cytokines (12). A complex collection including TGF-β, IL-1β, IL-6, IL-23, etc. affects the differentiation of Th17 cells (54, 89, 136). Specifically, TGF-β, IL-6 and IL-21 act together on immature T cells, inducing the expression of IL-1R and IL-23R and mediating the initial differentiation into Th17 cells (98, 137, 138). After that, IL-1β, IL-6 and IL-23 promote and stabilize the differentiation of Th17 cells through synergism (83). The secreted IL-21 then forms a positive feedback loop, exaggerating its own production (99). Meanwhile, there is accumulating evidence that genetic polymorphisms in these key regulators are associated with host susceptibility to periodontitis (47).

#### TGF-β

Although still under debate, the current view is that at initial differentiation stage, TGF-β drives CD4^+^ T cells to differentiate into Th17 cells and Treg, rather than Th1/2 cells, by inducing the expression of RORγt and FOXP3. Subsequent differentiation depends on the activation of the mutually antagonistic STAT3 (which promotes Th17 differentiation) and STAT5 (which promotes Treg differentiation) (11, 137–139).

TGF-β regulates Th17 cells by both canonical (small mother against decapentaplegic (SMAD)-dependent) and non-canonical (SMAD-independent) pathways (140, 141). Members of the SMAD family are the substrates of TGF receptor signaling and they decide the consequent effect. TGF-β receptor signaling regulates SMAD2 and SMAD3. Activated SMAD2/3 then combines with SMAD4, and they finally bind to DNA together to activate or repress Th17 cell transcription, which depends on the different phosphorylation states of SMAD2 and SMAD3 (140, 142). Phosphorylated SMAD2 positively regulates Th17 differentiation by STAT3 activation and the synergistical effect between STAT3 with RORγt. While unphosphorylated SMAD3 binds to RORγt and then inhibits its transcriptional activity (142). Recent studies found that SMAD4 itself does not directly regulate Th17 cell differentiation. Instead, SMAD4 interacts with other transcriptional modulators to perform regulatory functions. For example, SMAD4 recruits and mediates SKI which possesses the real suppressive effect in Th17 cell activation (143, 144). Additionally, TGF-β regulates Th17 cell differentiation through SMAD-independent pathways such as NF-κB pathways and MAPK pathways (140, 141). Recent studies on the effects of TGF-β on Th17 cells have concentrated more on the heterogeneity and plasticity of Th17 cells, which we describe below.

#### IL-1β, IL-6, and IL-23

IL-1β regulates Th17 differentiation through multiple mechanisms. ①IL-1β signaling positively regulates Th17 cell differentiation through the induction of interferon regulatory factor 4 (IRF4) (145, 146). ②IL-1β promotes Th17 differentiation by excising *FOXP3* exon 7 (147). ③IL-1β affects AKT-mTOR signaling pathway which is essential for the survival and proliferation of polarized Th17 cells. AKT, glycogen synthase kinase 3α (GSK3α) and IKKi form a complex in which IKKi is negatively regulated by GSK3α. IL-1β activates IKKi and impairs the function of GSK3α, thus leading to AKT-mTOR activation (148). ④IL-1β activates DCs and enhances IL-17 secretion by Th17 cells in a CD14-dependent manner (149). ⑤IL-1β downregulates SOCS3 to enhance the amplitude and duration of STAT3 phosphorylation induced by IL-6 and IL-23 (150). ⑥IL-1β synergizes with IL-6 to promote Th17 cell differentiation and proliferation through direct RORγT expression in CD4^+^ T cells (89). ⑦In the absence of IL-6, excessive IL-1 signaling enhances Th17 cell responses by downregulating TGF-β-induced Foxp3 expression (151).

IL-6 binds to IL-6R (composed of IL-6Rα and gp130) and phosphorylates the JAK family *via* gp130 which then activates STAT3, whereas suppressing the activation of STAT1 which would inhibit Th17 cell differentiation (152, 153). IL-6 also induces IL- 23R expression in naive T cells through the binding of STAT3 to *IL23r* locus, allowing IL23 to participate in late Th17 differentiation despite its initial absence due to the lack of IL-23R expressed on naive T cells (154). After IL-23R upregulation, the IL-23 signaling pathway activates STAT3 *via* the JAK family (155). IL-6 and IL-23-activated STAT3 up-regulates the expression of Th17 marker gene *Rorc*, producing RORγt that interacts with IRF4, BATF and other transcription factors to up-regulate the expression of Th17 cell lineage markers such as IL-17A, CCR6 (11, 156). STAT3 also regulates the expression of Th17 differentiation-related genes including *Il17a*, *Il17f*, *Il21* and *Il6ra* as well as cell survival and proliferation genes like *Bcl2*, *Fos* and *Jun (* 83, 156).

#### IL-2

IL-2 possesses a suppressive function in Th17 cell differentiation. IL-2 activates STAT5 and then enhances the expression of FOXP3, impeding the binding of STAT3 to the *Il17a* promoter and antagonizing transcription factors such as RORγt and Runt-related transcription factor (RUNX) 1 *via* JAK1/3 (157). In addition, higher FOXP3 mRNA expression was also speculated in periodontitis accompanied by increased RANKL and Th17-related genes mRNA levels, suggesting self-restraint of the host inflammatory response (158, 159). Low-dose IL-2 treatment is reported to be beneficial to the balance of Th17/Treg cells in other inflammatory diseases like SLE and arthritis (160, 161). However, it is also noted that IL-2 depletion resulted in higher levels of apoptosis in Th17, as low levels of IL-2 produced by Th17 cells mainly promote the expansion of Th17 cells (162). More experiments are needed to explore the mechanisms underlying the effect of IL-2 on Th17 cell differentiation.

### Heterogeneity and Plasticity of Th17 Cells

Intriguingly, not all Th17 cells boost inflammation and not all Th17 cells exacerbate inflammation through IL-17A. The molecular underpinning for such biological behaviour is the heterogeneity and plasticity of Th17 cells. Heterogeneity means that different Th17 subsets display different levels of pathogenicity, namely immunoregulatory IL-10^+^ Th17 cells and pro-inflammatory Th17 cells (163, 164). Plasticity means that Th17 cells possess the ability to trans-differentiate into phenotypes other than IL-17^+^ Th17 cells and express cytokines typical of other lineages (164).

Researchers believe that it is the cytokine milieu that determines Th17 cell phenotype. As previously stated, pathogenic Th17 cells are induced in IL-1β, IL-6 and IL-23 condition or in IL-6 and TGF-β3 condition (136, 165). However, non-pathogenic Th17 cells are differentiated by TGF-β1 or IL-6 (136, 166). Single-cell RNA sequence technology unveiled the transcriptional signatures of non-pathogenic and pathogenic Th17 cells. Pathogenic Th17 cells express more pro-inflammatory genes module including *Il23r, IL22, Il17a*, and *Il17f*, while non-pathogenic Th17 cells upregulate the expression of immune suppressive genes like *Il10, Il4, Ahr* and *c-maf (* 167, 168). Their differences in pathogenicity explain opposite results shown in clinical trials targeting IL-17A signals in different diseases (115, 169, 170). Of note, c-Maf is indicative of pathogenicity because it regulates IL-10^+^ Th17 cell transcription *via* the MAPK pathway (163, 171). In other words, pathogenetic Th17 cells expresses less c-Maf compared with non-pathogenetic Th17 cells.

Th17 cells could also be inverted into an anti-inflammatory phenotype termed Th17-derived Tr1-like cells (exTH17) cells, which is induced by TGF-β1 *via* SMAD3 and aryl hydrocarbon receptor (AHR) (172). The inversion is related to Th17 cells plasticity, as evidences accumulate that Th17 cells seem to be unstable terminally differentiated cells (173, 174). Th17 cells are now considered to have the potential for phenotypic trans-differentiation into mainly Th17/Th1 cells, Th17/Th2 cells and even Th17/Treg cells, which may be altered by co-expression of CD4^+^ T cell lineage transcription factors (164, 175).

Recently, new breakthroughs have been made in the study of Th17 cells plasticity. Th17 cells may transform into Th1-like CXCR3^+^ Th17 cells (Th17.1 cells) under the regulation of IL-12 and IL-23. Interestingly, the absence of TGF-β1 not only promotes pathogenic Th17 cells, but also upregulates T-bet expression and shows much higher plasticity in transitioning into Th1-like Th17 cells, because early TGF-β1 suppression on T-bet is relieved (176). The expression of RUNX 1 in Th17 cells could be enhanced by IL-12 stimulation and then binds to the *Ifng* locus in a T-bet-dependent manner, thus showing a phenotype that secretes IFN-γ (177). Namely, classical Th17 cells mainly express IL-17A, while Th17.1 cells develop into IL-17A^+^ IFN-γ^+^ cells or IL-17A^-^ IFN-γ^+^ cells (174).

Th17.1 cells show stronger pro-inflammatory properties, supported by higher proliferation ability in response to T cell receptor (TCR) signals, higher GM-CSF, CCL20 and IL-22 production (178, 179). A latest study, tracking the plasticity of Th17 cells in periodontitis model, suggested that the transformation of classical Th17 cells to ex-Th17 cells occurs during the conversion from acute inflammation to chronic inflammation (180). The dysbiosis caused by *P. gingivalis* may drive this transition through the increment of IL-17A in the early stage and the dominant expression of IFN-γ in the later stage (180).

### Current and Potential Therapies Targeting Th17 in Periodontitis

Given the essential role of Th17 cells in periodontal inflammation and alveolar bone loss, it is conceivable that targeting Th17 cells and related key molecules is of great potential. Here we briefly describe some experimental therapies focusing on Th17 modulators, along with their results.

IL-17 acts as the main cytokine in the pathogenesis of periodontitis, and intervention experiments targeting IL-17 achieved positive results. Suppressed IL-17 expression significantly reduced alveolar bone resorption occurs in mice (17). Inhibiting RORγt and then down-regulating IL-17 expression *via* GSK805 or curcumin attenuated alveolar bone loss (17, 82). Beyond periodontitis, therapies targeting IL-17/IL-17R to treat autoimmune diseases such as psoriasis have achieved positive results (181). In a phase III, randomized double-blind placebo-controlled study using Brodalumab (a monoclonal antibody against IL-17RA) on moderate-to-severe plaque psoriasis, more than 70 percent of patients achieved a 75% reduction in psoriasis area severity index at 12 weeks, which is much higher than the placebo group (182). As previously mentioned, however, it was also reported that IL-17RA^KO^ mice exhibited profound alveolar bone destruction for impaired chemokine expression and neutrophil migration (79). More studies targeting IL-17/IL-17R are required to investigate the exact mechanisms. Also, derived clinical trials aiming at periodontitis are essential for verifying the therapeutic effects.

IL-6 and IL-23 support the survival and expansion of Th17 cells (154). Tocilizumab (TCZ) is a recombinant humanized monoclonal antibody which binds to human IL-6R and inhibits IL-6 signaling (183). TCZ treatment alleviated periodontal inflammation in patients, compared with those without TCZ therapy (184–186). As to IL-23, a case report claimed that systemic usage of ustekinumab, a monoclonal antibody blocking the p40 subunit of IL-23, resolved inflammatory lesions in a patient with LAD-I (187). JAK is a pathway downstream of IL-6. Patients who received tofacitinib, an inhibitor for JAK, also showed reduced periodontal inflammation (188).

MicroRNAs (miRs) act as vital regulators of Th17 differentiation and periodontal inflammation (189, 190). MiR-155 up-regulates Th17 responses and enhances osteoclastogenesis, while exosomal miR-155-5p from periodontal ligament stem cells (PDLSCs) could be transferred into CD4^+^ T cells and then decrease RORC expression, alleviating inflammatory microenvironment (191, 192). More studies are needed to explore the exact effects and mechanisms of miRs on regulating Th17 differentiation in periodontitis.

Some studies comment deubiquitylating enzymes (DUBs) as potential modulators of periodontitis progression because DUBs modulate IL-17 signaling by TRAFs. In particular, A20 is a protein that possesses DUB and regulates Th17 differentiation and IL-17 function (27). As several studies have demonstrated the anti-inflammatory effects of A20 especially *via* regulating Th17 differentiation, we consider A20 as a promising therapeutic target for periodontitis treatment (193–195). Hereafter, we focus on recent advances and regulatory mechanisms of A20 in modulating Th17 and IL-17.

## A20: Novel Therapeutic Target by Th17 and IL-17 Modulation

Ever since it was first identified in 1990 as an inhibitor of NF-κB pathway in response to TNF, A20 has established its role as a potent anti-inflammatory molecule, mainly attributed to its ubiquitin-editing function (25). On the N-terminal of A20, OTU domain deubiquinates K48 and K63-linked ubiquitin chains, the former target substrates for proteasomal degradation, whereas the latter often save targets from degradation (27). On its C-terminal, ZnF4 interacts with K63-linked ubiquitin chains and ZnF7 binds with M1-linked ubiquitin chains (196, 197). As a ubiquitin-editing enzyme, A20 restricts excessive inflammation mainly by deubiquinating TRAF6, thereby blocking NF-κB pathway and arresting immune responses (26). In recent years, A20 has displayed pleiotropic effects in cell death, tumorigenesis and autoimmune diseases (29–31). Hereinbelow, we discuss the role of A20 in Th17 cell differentiation and IL-17 function.

### A20 Inhibits Th17 Cell Expansion Through Diminished IL-6 Production

It is established that Th17 differentiation is dependent on cytokines like IL-6, IL-23, IL-17, the production of which relies on NF-κB signaling pathway that exaggerates inflammatory signals. As a negative regulator of NF-κB signaling pathway, the inhibitory role of A20 on cytokine production account for the increase of Th17 cells observed in A20-deficient models.

NF-κB pathway is a ubiquitous signaling pathway that modulates cell proliferation, immune responses, necroptosis, and so forth. Considerable attention has been paid to its role in tumorigenesis and inflammatory diseases (198, 199). After being initiated by pathogens, pro-inflammatory cytokines and others, IKK complex phosphorylates IκB and the latter undergoes proteasomal degradation (26). The subsequent translocation of NF-κB into the nucleus then activates NF-κB-related genes and produces, including but not limited to, pro-inflammatory cytokines such as IL-1, IL-6 and TNF, growth factors, chemokines, as well as inhibitors of NF-κB pathway like IκBα and A20 to avoid excessive inflammation (26).

A20 deubiquinates IKKγ (also known as NF-κB essential modulator, NEMO) and TRAF6, an E3 ubiquitin ligase that is vital in the activation of IKK complex (200–202). The activation of IKK complex terminates NF-κB pathway and dampens NF-κB-mediated inflammation, exaggerating inflammation and elaboration of pro-inflammatory cytokines like IL-6, TNF. By the same token, A20 also dampens MAPK (especially JNK) activation through TRAF6 deubiquitylation (27, 203). Therefore, A20 depletions in human macrophage-like cells (THP-1) and in mice bone marrow derived macrophages lead to increase in cytokine production *in vitro (* 194). Consistently, partial loss of A20 in mice show severer alveolar bone loss, more infiltration of immune cells, more pro-inflammatory cytokines including IL-6, IL-23, IL-17, and these mice display prolonged NF-κB activation (194).

The postulation that deficiency of A20 leads to a plethora of inflammatory IL-6 that drives Th17 differentiation has been confirmed in an arthritis-related study where A20 inhibits Th17 cell differentiation through IL-6 in mice lacking A20 in their bone marrow mesenchymal stem cells (BM-MSCs) (138, 204). The fact that A20 restricts IL-17 signaling and the concomitant decrease of its own implicates a negative feedback loop that maintains an equilibrium between inflammatory responses and homeostasis, avoiding excessive tissue damage and autoimmune disorders.

### ZnF7 Motif in A20 Restricts Th17 Cell Proliferation

Hereinbefore, A20 possesses a ZnF7 motif on its C-terminal that is capable of binding with M1-linked ubiquitin chains (28). Research has shown that A20 represses inflammatory diseases through its ZnF4 and ZnF7 motif synergistically in a non-catalytic way (205). Mouse model A20^ZF7/ZF7^, harboring a point mutation in C103 that disabled its ZnF7 motif, displayed an elevation in IL-17-expressing T cells, compared to its wild type littermates (205). As an array of studies showed that commensal bacterium is crucial to Th17 in arthritis, gastrointestinal tract and skin, A20^ZF7/ZF7^ were further bred in germ-free conditions this time to interrogate the relationship between Th17 cell expansion and commensal microbe colonization, however results did not show any causal relationship (36, 205–207). Th17 cell proliferation in the human gingival oral mucosal barrier is also independent of commensal bacterium (37). Therefore, ZnF7 motif might be a plausible target for therapeutic interventions although the underlying mechanism warrants further investigation at molecular and cellular levels.

### A20 Binds to the C-Terminal of IL-17RA and Downregulates IL-17 Signaling

The IL-17 family includes ligands IL-17A to IL-17F that bind to IL-17RA to IL-17RE. IL-17 receptor is a heterodimer composed of IL-17RA and IL-17RC, both of which contains a SEFIR domain that is conserved in the IL-17R family (95). Upon IL-17 activation, SEFIR domain binds with adaptor protein Act1, which also contains a SEFIR domain, through homotypic interactions, thereafter, serving as a docking site for TRAF proteins (208). Specifically, Act1 recruits TRAF6 and triggers K63- ubiquitylation with the help of E3 ligase activity of Act1 (203, 209). Ubiquinated TRAF6 leads to the activation of IKK complex, subsequent phosphorylation and degradation of IκB pave the way for the initiation of canonical NF-κB pathway and promotes transcription of pro-inflammatory proteins such as cytokines, chemokines, and A20 (210). On the C-terminal of IL-17RA, CBAD is indispensable to the activation, translation and phosphorylation of C/EBPβ and negatively controls IL-17-induced signaling (27). CBAD contains a TRAF consensus site that helps TRAF3 replace Act1, ultimately mitigating IL-17R signaling.

**Figure 4:**
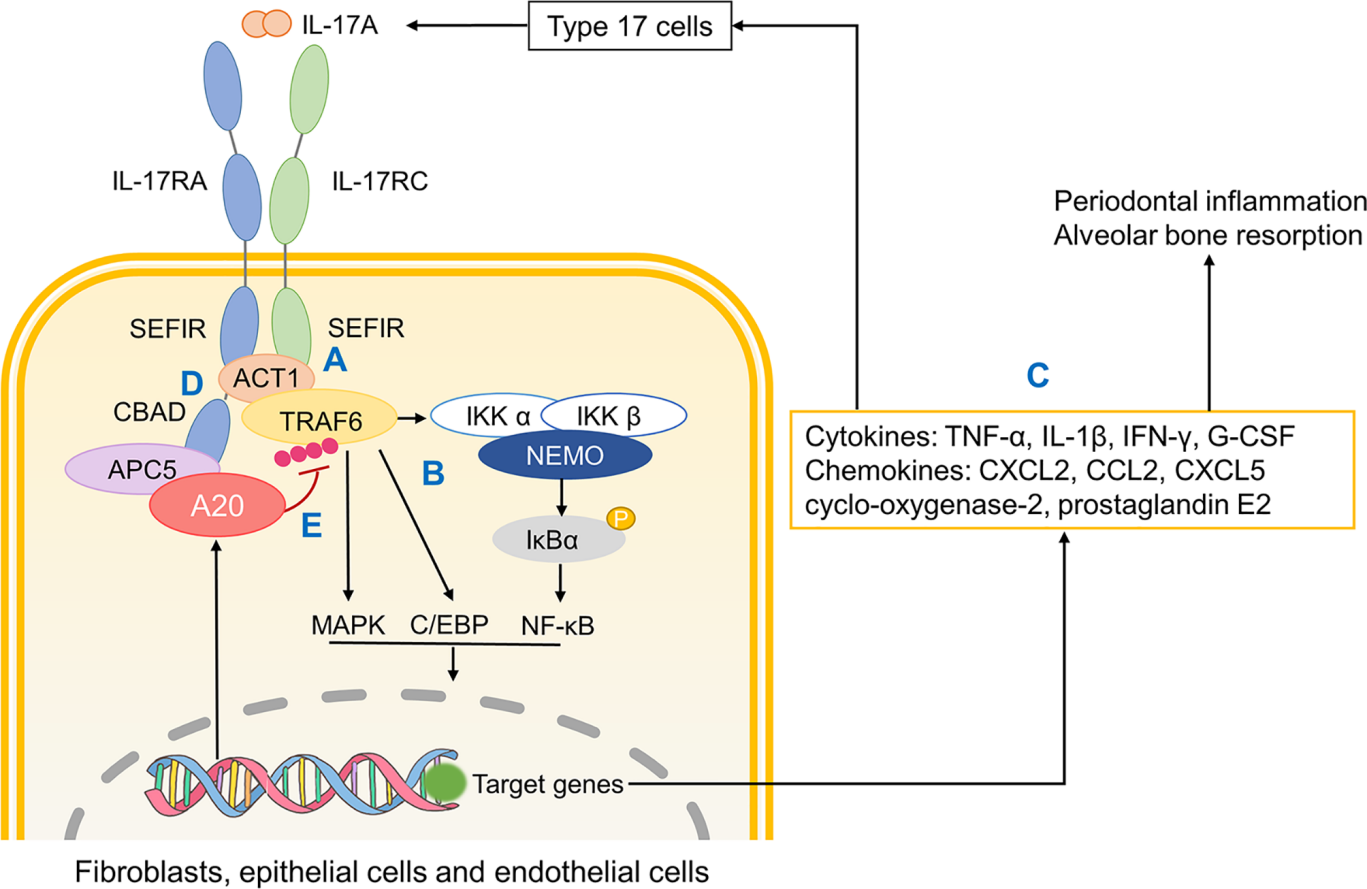
The role of A20 in IL-17A signaling. **(A)** Upon IL-17A activation, two SEFIR domains respectively in IL-17RA and IL-17RC bind to Act1. Then Act1 recruits and ubiquinates TRAF6. **(B)** Ubiquinated TRAF6 activates downstream MAPK, C/EBP and NF-κB pathways. **(C)** Related gene transcription promotes the production of cytokines, chemokines that not only promote inflammatory responses and osteoclastic resorption but also further recruit monocytes and macrophages. **(D)** CBAD in IL-17RA helps TRAF3 replace Act1, which terminates IL-17R signaling. **(E)** A20 binds to the inhibitory domain CBAD through APC5 thus ceasing downstream pathways. SEFIR, SEF/IL-17R; TRAF, tumour necrosis factor receptor-associated factor; MAPK, mitogen-activated protein kinase; NF-κB, nuclear factor-κB; CBAD, CCAAT/enhancer binding protein β (C/EBP β) activation domain; APC5, anaphase promoting complex protein 5.

It is substantiated that A20 binds to CBAD in IL-17RA, albeit not SEFIR domain, through anaphase promoting complex protein 5 (AnapC5 or APC5) which is known for its role in regulating cell cycle (211) (**Figure 4**). During this process, APC5 serves as an adaptor protein that facilitates the binding of A20 to inhibitory domain CBAD in IL-17RA and this interaction between A20 and CBAD ceases IL-17 receptor signaling.

### A20 Inhibits the Maturation of IL-1β and Hinders Th17 Recruitment

Apart from being the end-product of Th17 cell secretion, IL-17 is capable of synergizing with IL-1β to promote CCL20 in human fibroblasts, consequently recruiting more Th17 cells and forms a feedback loop (212). As a matter of fact, Th17 cells also induces CCL20 and exaggerates its own pro-inflammatory effects (212). A20 mediates this feedback negatively by restricting IL-1β.

Unlike in GI tract and skin, the differentiation of Th17 cells in the oral cavity is independent of IL-1β, but IL-1β still appears critically positioned in the etiology of periodontitis, especially in immunomodulation and bone resorption (37, 213). IL-1β activates endothelial cells and promotes the adhesion of eosinophils, which exaggerates inflammation (214). IL-1β promotes the production of RANKL, which is vital to bone resorption as stated before (215). IL-1β also upregulates the formation and bioactivity of osteoclasts eventually leading to alveolar bone resorption (216). The secretion of IL-1β could be divided into two steps. Firstly, in response to microbial signals, pattern recognition receptors (PRRs), normally containing pyrin and/or caspase activation and recruitment domain (CARD), secrete pro-proteins like pro-IL-1β and pro-IL-18 through NF-κB pathway (217). Secondly, PRRs recruit the cysteine protease caspase-1 directly or indirectly through apoptosis-associated speck protein containing a CARD (ASC) (217). Caspase-1 cleaves inactive pro-proteins proteolytically and confer them with bioactivity but this requires a prior activation signal (218). An array of studies has confirmed that this activation signal is provided by inflammasomes, a multiprotein signaling complex assembled by members of the nucleotide-binding domain (NOD) and leucine-rich repeat containing (LRR) (NLR) family or the pyrin and HIN-domain (PYHIN) family (219).

In the case of IL-1β, a member of NLR family called NLR and pyrin domain (PYD)-containing protein 3 (NLRP3), along with caspase-1 and ASC, assemble into NLRP3 inflammasome (220). The bioactivity of NLRP3 inflammasome calls for two steps, priming and activation. The priming of NLRP3 inflammasome requires microbial signals like NF-κB-dependent TLRs or TNF but there still lacks a unified theory on the secondary activation signal (219). Current studies suggest that the rise in extracellular ATP activates P2X7 and this triggers K^+^ efflux, eventually activating NLRP3 inflammasome (221).

As a pathological contributor to oral diseases, NLRP3 inflammasome activates caspase-1 and caspase-1 matures pro-inflammatory cytokines IL-1β and IL-18 (222). Meanwhile, NLRP3 also induces pyroptosis, a type of inflammation-associated cell death, by cleaving the N-terminal of gasdermin D (GSDMD) and forming a pore on the cell membrane (223). NLRP3 also promotes alveolar bone resorption through osteoclast differentiation (224). The level of NLRP3 inflammasome mRNA and its related proteins are increased in periodontitis and gingivitis, which corroborates a negative role of NLRP3 in the oral cavity (225).

**Figure 5:**
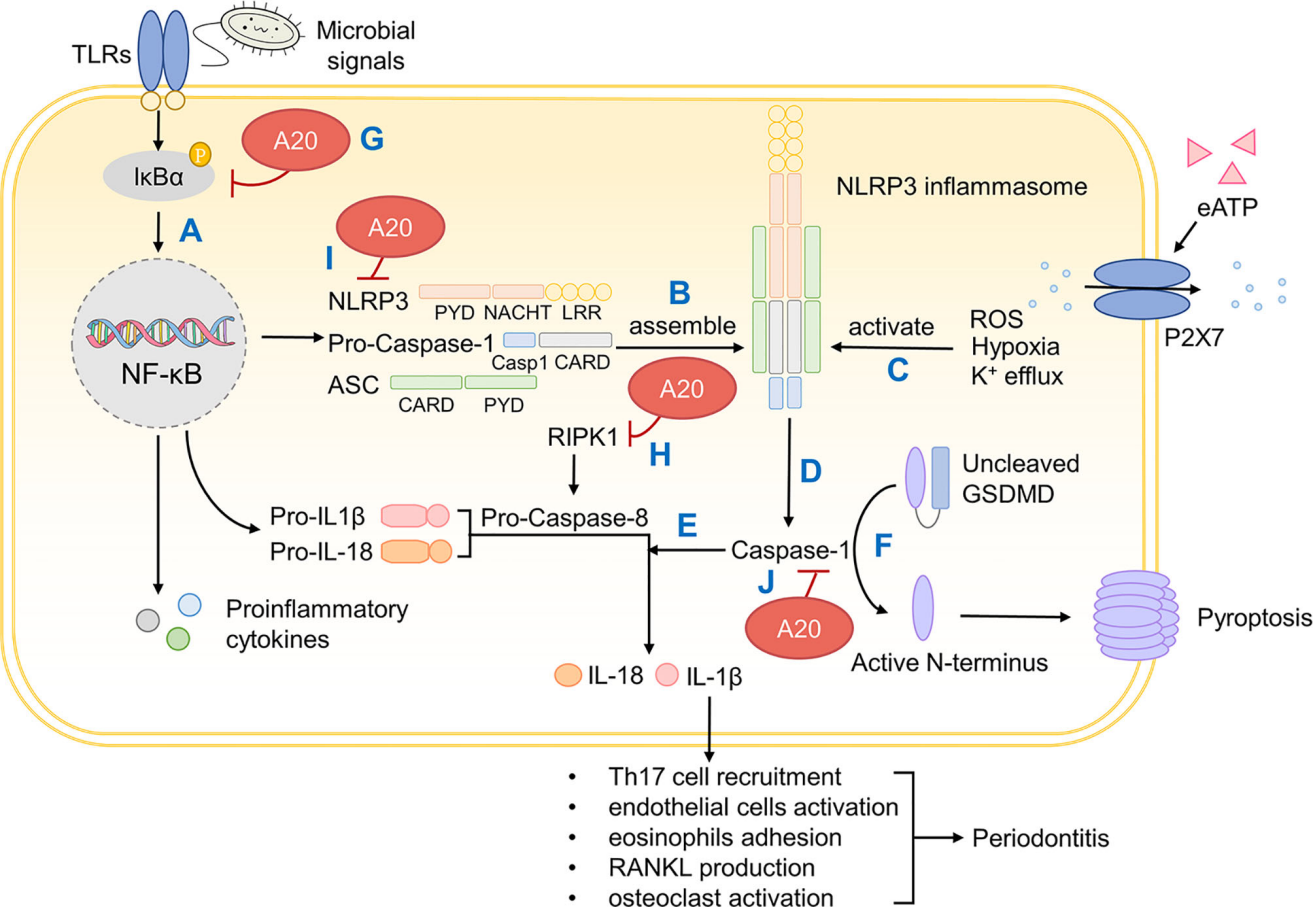
A20 inhibits IL-1β production and downstream periodontal inflammation. **(A)** On microbial activation, PRRs containing CARD induces pro-IL-1β and pro-IL-18 production through NF-κB pathway. **(B)** NLRP 3 inflammasome is assembled by NLRP3, pro-Caspase-1 and ASC. **(C)** NLRP3 inflammasome is primed by microbial signals and activated by ROS, hypoxia and K^+^ efflux. **(D)** Mature NLRP3 inflammasome confers Caspase-1 with bioactivity. **(E)** Caspase-1 cleaves and activates pro-IL-1β and pro-IL-18 that induces inflammation. **(F)** Caspase-1 also cleaves GSDMD whose active N-terminus forms a pore on the cellular surface and causes pyroptosis. **(G)** A20 inhibits NF-κB signaling through IκBα. **(H)** A20 degrades RIPK1, impedes pro-Caspase-8 production and thus inhibits IL-1β production. **(I)** A20 restricts NLRP3 function through caspase1-caspase8- RIPK1-RIPK3 complex. **(J)** A20 inhibits caspase-1 dependent pyroptosis. PRRs, pattern recognition receptors; CARD, caspase activation and recruitment domain; NLRP3, NOD (nucleotide oligomerization domain)-, LRR (leucine-rich repeat)-, and PYD (pyrin domain)-containing protein 3; ASC, apoptosis-associated speck protein containing a CARD; ROS, reactive oxygen species; GSDMD, gasdermin D; IκBα, inhibitor of NF-κB alpha; RIPK, receptor-interacting protein kinase.

Considerable research has focused on the inhibitory role of A20 in NLRP3 inflammasome maturation (**Figure 5**). First of all, A20 downregulates NF-κB signaling pathway, which directly limits the production of pro-proteins and microbial components required for the priming of NLRP3 inflammasome (202). Besides, caspase-8 in A20-deficient cells shows elevated activity and cleaves in more pro-IL-1β, suggesting a negative role of A20 in mediating caspase-8 and IL-1β production (217). Also, A20 restricts NLRP3 function through caspase1-caspase8- receptor-interacting protein kinase (RIPK)1-RIPK3 complex (217). Apart from that, A20 inhibits caspase-1-dependent pyroptosis (226). Although the underlying mechanism remains incompletely understood, this still substantiates the regulative role of A20. In summary, A20 inhibits the production of IL-1β thereby mitigating downstream inflammatory responses and bone resorption.

## Conclusion

Periodontitis is a multifactorial chronic oral disease affecting a considerable proportion of the world population. Over the years, evidence has moved from minimal to substantial that periodontitis is closely related to an array of systematic diseases like diabetes, obesity, hypertension, and especially rheumatoid arthritis. Hence, superior periodontal treatments may lead to improved overall health conditions. Although modern surgical and non-surgical treatments against periodontitis have helped patients to some extent, the underlying mechanism has not been fully revealed. Here we conclude the pathogenesis of periodontitis and the essential role of Th17 cells in it. We also summarized some modulators of Th17 cells for they could be future therapeutic targets.

However, current understanding of Th17 cells and periodontitis is far from enough. For example, anti-IL-17A antibody alone could not alleviate periodontitis because of the concomitant rise in GM-CSF. Would the addition of anti-GM-CSF antibody be helpful? Also, the heterogeneity and plasticity of Th17 cells have hinted us that therapies targeting Th17 cells need revision. Does ex Th17 exhibit bioactivities that we are current unaware of? Is it possible that we can create a cytokine milieu that converts pathogenic Th17 cells to non-pathogenic ones? These questions await further explanation.

Given the outstanding performance of A20 in restricting Th17 cells, we anticipate that A20 may be a potential target in restricting periodontal inflammation and bone resorption but there are still many open questions as to whether there are more explanations for the interplay between A20 and Th17 cell expansion. For example, the cellular mechanism through which ZnF7 motif in A20 restricts Th17 differentiation remain ill-defined and the interplay between IL-17/IL-23 axis and A20 is yet not understood and this calls for further exploration into the anfractuous immune system.

## Author Contributions

NH, HD, and YL wrote the manuscript. BS reviewed the manuscript. All authors contributed to the article and approved the submitted version.

## Funding

This study was funded by the National Natural Science Foundation of China, Grant/Award Numbers: 81972193 and 81702271; the Department of Science and Technology of Sichuan Province, Grant/Award Number: 2019YJ0041; the Scientific Research Foundation for Recruited Talents, West China Hospital of Stomatology Sichuan University, Grant/Award Number: QDIF2019-1.

## Conflict of Interest

The authors declare that the research was conducted in the absence of any commercial or financial relationships that could be construed as a potential conflict of interest.

## Publisher’s Note

All claims expressed in this article are solely those of the authors and do not necessarily represent those of their affiliated organizations, or those of the publisher, the editors and the reviewers. Any product that may be evaluated in this article, or claim that may be made by its manufacturer, is not guaranteed or endorsed by the publisher.
